# Primary Diffuse Large B-cell Lymphoma of the Terminal Ileum Presenting With Bowel Perforation and an Acute Abdomen in an Elderly Patient

**DOI:** 10.7759/cureus.109521

**Published:** 2026-05-23

**Authors:** Yanina Nikolaus, Saadia Haleema

**Affiliations:** 1 Pathology, Marshall University Joan C. Edwards School of Medicine, Huntington, USA

**Keywords:** acute abdomen, bowel perforation, diffuse large b-cell lymphoma, dlbcl, gastrointestinal lymphoma, immunohistochemistry, intestinal lymphoma, non-hodgkin lymphoma, small bowel lymphoma, terminal ileum

## Abstract

Diffuse large B-cell lymphoma (DLBCL) is the most common subtype of non-Hodgkin lymphoma; however, primary involvement of the small intestine is uncommon. Acute bowel perforation is a rare and life-threatening presentation that may mimic more common causes of acute abdomen. We report the case of a 78-year-old woman who presented with worsening abdominal pain, nausea, vomiting, and obstipation. Computed tomography demonstrated pneumoperitoneum concerning for hollow viscus perforation. Exploratory laparotomy revealed a perforated terminal ileal mass, and segmental small bowel resection was performed. Histopathologic evaluation demonstrated diffuse transmural infiltration by large atypical lymphoid cells with diffuse CD20 positivity and MUM1 nuclear positivity, consistent with DLBCL, non-germinal center B-cell subtype by the Hans algorithm (CD10-negative, BCL6-positive, MUM1-positive). The postoperative course was complicated by sepsis, respiratory failure, and prolonged ileus. This case highlights the importance of considering primary gastrointestinal lymphoma in the differential diagnosis of bowel perforation and acute surgical abdomen, particularly in elderly patients presenting emergently.

## Introduction

Diffuse large B-cell lymphoma (DLBCL) is the most common subtype of non-Hodgkin lymphoma; however, primary involvement of the small intestine is uncommon [1]. The GI tract represents the most frequent extranodal site of lymphoma involvement, with the stomach being the most commonly affected location, whereas primary small bowel lymphomas are considerably less frequent [2,3]. Among small intestinal sites, the terminal ileum is particularly susceptible because of its abundant lymphoid tissue and Peyer’s patches [2,3]. Gastrointestinal lymphomas frequently present with nonspecific symptoms, including abdominal pain, weight loss, gastrointestinal bleeding, nausea, vomiting, or obstructive symptoms, which may delay diagnosis until advanced disease or acute complications develop [2-4]. Bowel perforation is a rare but life-threatening presentation associated with significant morbidity and mortality [4,5]. In emergency surgical settings, intestinal lymphoma may clinically and radiographically mimic more common entities, such as ischemic bowel disease, inflammatory bowel disease, infectious enterocolitis, or adenocarcinoma, creating substantial diagnostic challenges. Early recognition of this entity is important, as delayed diagnosis and treatment may adversely affect outcomes, particularly in elderly patients presenting with an acute abdomen [1,2]. Although gastrointestinal lymphomas may present with nonspecific abdominal symptoms, presentation with frank bowel perforation remains uncommon [4,5].

## Case presentation

A 78-year-old woman with a medical history significant for coronary artery disease, transcatheter aortic valve replacement, atrioventricular block status post pacemaker placement, chronic kidney disease, hypertension, hypothyroidism, and macular degeneration presented as a transfer from an outside facility with a two-day history of worsening abdominal pain associated with nausea, vomiting, and obstipation. She denied tobacco, alcohol, or illicit drug use. Laboratory evaluation demonstrated marked leukocytosis and mild lactic acidosis. Initial laboratory findings are summarized in Table 1. A prior computed tomography angiography study performed approximately one year before presentation demonstrated no bowel wall thickening, intra-abdominal mass, fluid collection, or lymphadenopathy (Figure 1). At presentation, abdominal radiography demonstrated pneumoperitoneum concerning for perforated viscus (Figure 2). CT imaging of the abdomen and pelvis performed at the outside facility demonstrated pneumoperitoneum with suspected perforation involving the distal small bowel and associated inflammatory change within the right lower quadrant, concerning for hollow viscus perforation and an acute surgical abdomen.

**Table 1:** Initial laboratory findings at presentation.

Laboratory test	Patient value	Reference range
WBC count	14.44 × 10³/µL	4.0-11.0 × 10³/µL
Hemoglobin	11.5 g/dL	12.0-16.0 g/dL
Platelet count	154 × 10³/µL	150-400 × 10³/µL
Sodium	135 mmol/L	136-145 mmol/L
Potassium	3.7 mmol/L	3.5-5.1 mmol/L
Carbon dioxide (CO₂)	21 mmol/L	22-29 mmol/L
Blood urea nitrogen	33 mg/dL	7-20 mg/dL
Creatinine	1.10 mg/dL	0.6-1.3 mg/dL
Glucose	186 mg/dL	70-99 mg/dL
Calcium	7.6 mg/dL	8.5-10.5 mg/dL
Albumin	2.0 g/dL	3.5-5.0 g/dL
Total protein	4.9 g/dL	6.0-8.3 g/dL
Alkaline phosphatase	123 U/L	44-147 U/L
Lactic acid	2.1 mmol/L	0.5-2.0 mmol/L

**Figure 1:**
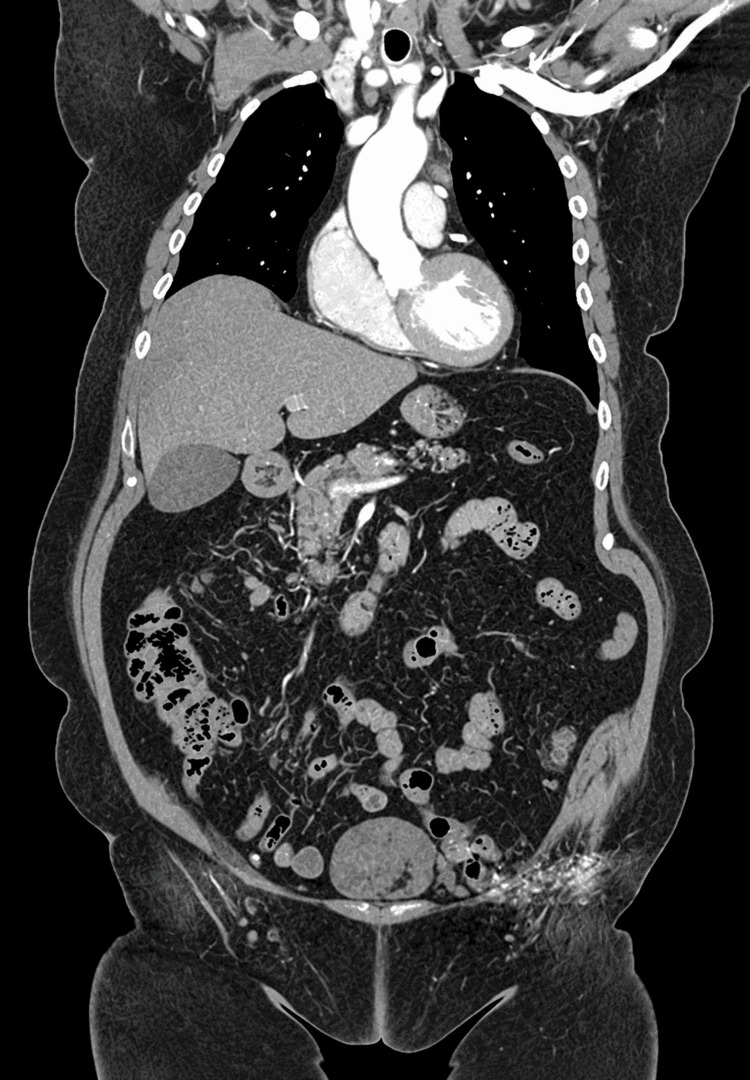
Computed tomography angiography performed approximately one year before diagnosis, demonstrating no radiologic evidence of bowel wall thickening, intra-abdominal mass, or lymphadenopathy.

**Figure 2:**
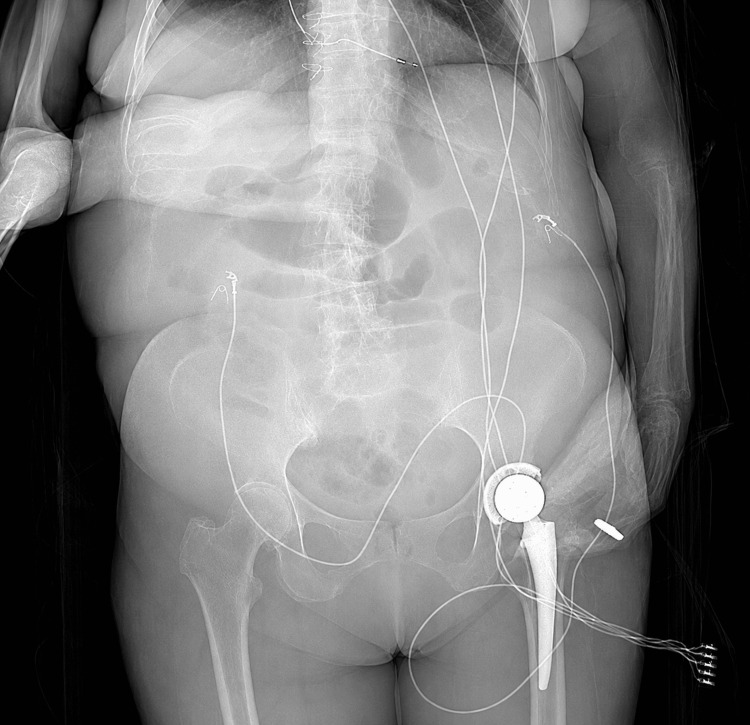
Abdominal radiograph obtained at presentation demonstrating pneumoperitoneum, concerning for perforated viscus.

Exploratory laparotomy revealed gross enteric contamination within the pelvis and a blow-out perforation involving greater than 70% of the terminal ileal wall, approximately 5 cm proximal to the ileocecal valve, with associated necrotic bowel edges and pelvic abscess formation. The affected bowel segment was adherent to the posterior uterus. Segmental small bowel resection was subsequently performed, with temporary abdominal closure and planned delayed reconstruction because of extensive intra-abdominal contamination.

Pathologic examination of the resected bowel identified a 3.2 cm tan-white mass centered within the terminal ileum, with associated bowel wall necrosis and perforation. The lesion involved the mucosa, submucosa, and muscularis propria without definite serosal or mesenteric fat involvement. Proximal, distal, and mesenteric soft tissue margins were negative for lymphoma. One regional lymph node was identified and was negative for lymphoma (0/1). No lymphovascular or perineural invasion was identified.

Histologic evaluation demonstrated extensive infiltration of the bowel wall by sheets of large atypical lymphoid cells associated with necrosis (Figure 3). High-power examination showed large pleomorphic lymphoid cells with vesicular chromatin, prominent nucleoli, and increased mitotic activity (Figure 4). Immunohistochemical studies demonstrated diffuse strong positivity for CD45, CD20 (Figure 5), PAX5, BCL6, BCL2, MUM1 (Figure 6), and p53. The neoplastic cells were negative for CD3, CD5, CD10, CD30, CK7, CK20, CDX2, synaptophysin, chromogranin, INSM1, and AE1/AE3. The Ki-67 proliferation index was approximately 60%.

**Figure 3:**
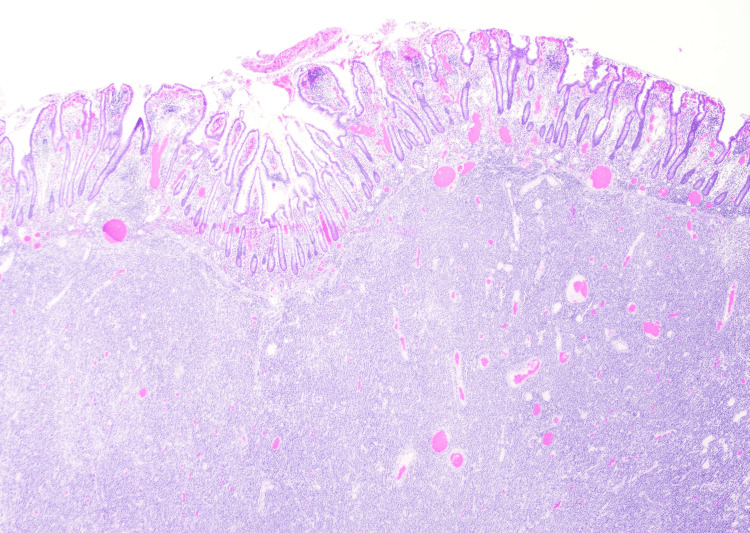
Low-power H&E-stained photomicrograph demonstrating extensive bowel wall infiltration by atypical lymphoid cells with associated necrosis in the terminal ileum (40×).

**Figure 4:**
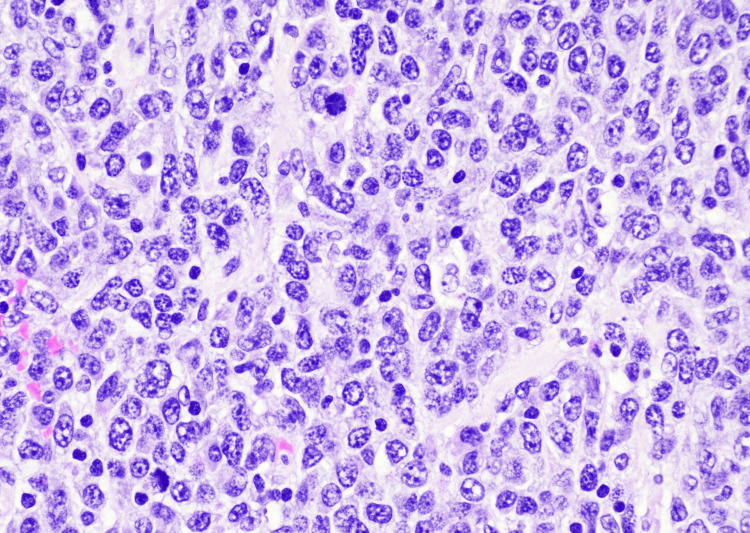
High-power H&E-stained photomicrograph showing large atypical lymphoid cells with vesicular chromatin and prominent nucleoli (400×).

**Figure 5:**
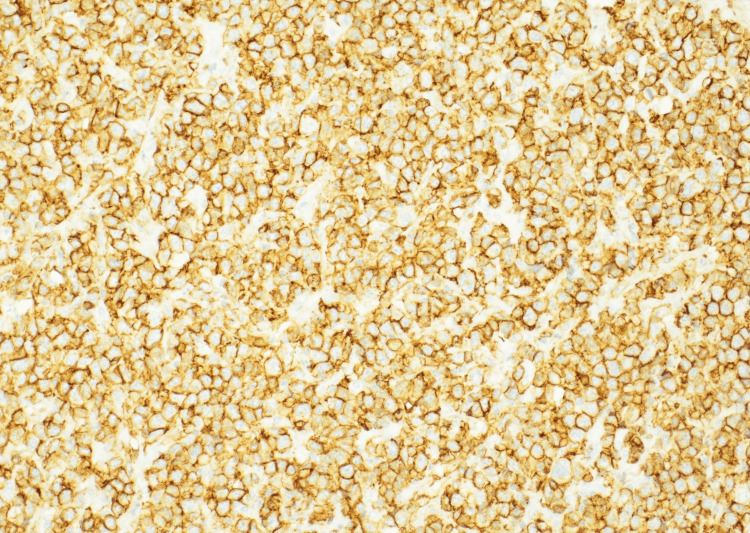
CD20 immunohistochemical stain demonstrating diffuse strong membranous positivity in neoplastic lymphoid cells (200×).

**Figure 6:**
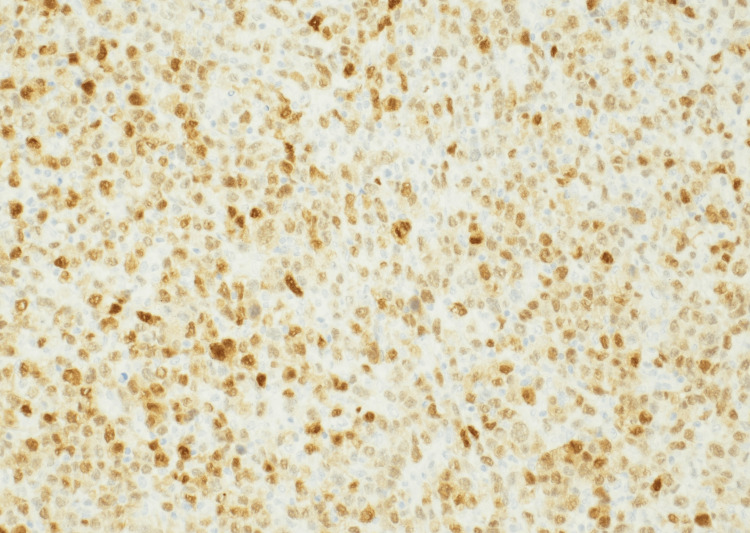
MUM1 immunohistochemical stain showing nuclear positivity in tumor cells, supporting classification as non-germinal center subtype diffuse large B-cell lymphoma in the appropriate immunophenotypic context (200×).

Based on the Hans algorithm, the immunophenotypic findings supported DLBCL, non-germinal center B-cell phenotype (CD10-negative, BCL6-positive, MUM1-positive). Mismatch repair protein expression was retained, with intact MLH1, PMS2, MSH2, and MSH6 expression.

Fluorescence in situ hybridization studies were negative for rearrangements involving MYC, BCL2, and BCL6, with no evidence of high-grade B-cell lymphoma with MYC and BCL2 and/or BCL6 rearrangements (“double-hit lymphoma”). Copy number gains involving BCL6, MYC, and BCL2 were identified; however, these findings were nonspecific and of uncertain clinical significance.

Follow-up fluorodeoxyglucose PET/CT performed approximately five months after surgery demonstrated a markedly hypermetabolic, partially necrotic right hemipelvic mass with a maximum standardized uptake value of 24.8 and associated hydronephrosis, concerning for persistent or recurrent disease (Figure 7).

**Figure 7:**
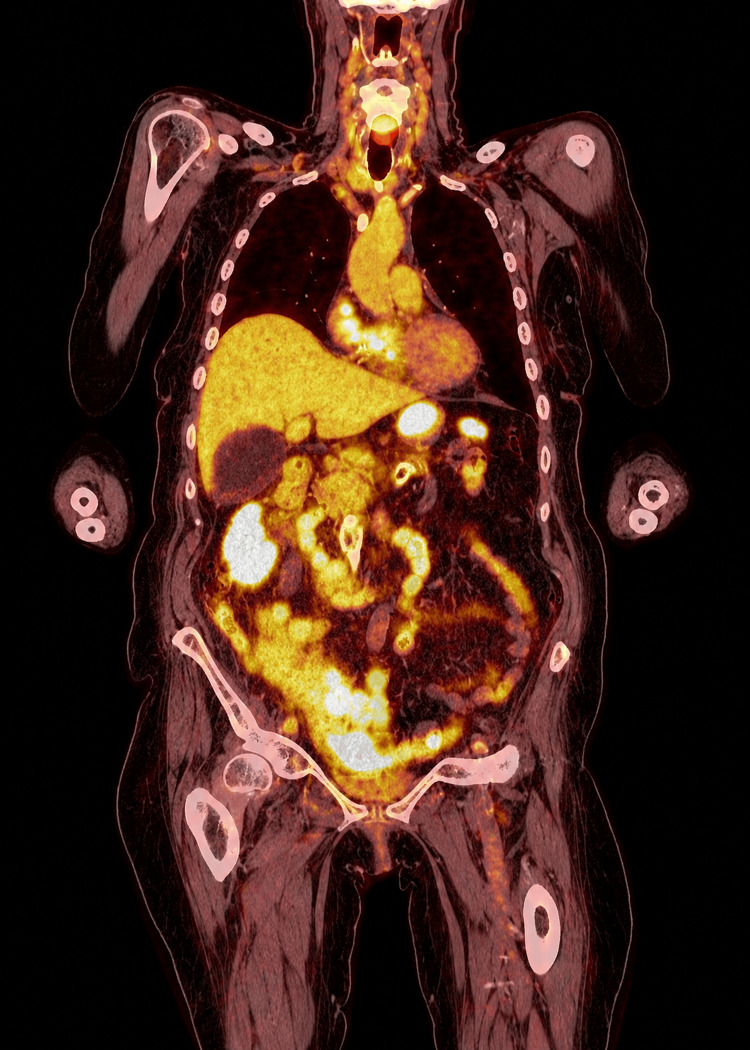
Coronal fluorodeoxyglucose PET/CT image demonstrating a markedly hypermetabolic, partially necrotic right hemipelvic mass with associated hydronephrosis, concerning for persistent or recurrent diffuse large B-cell lymphoma.

Differential diagnosis

The differential diagnosis included primary intestinal adenocarcinoma, ischemic enteritis with perforation, inflammatory bowel disease, infectious enterocolitis, and primary gastrointestinal lymphoma. The diffuse transmural proliferation of large atypical lymphoid cells and the immunophenotypic profile, including diffuse CD20 positivity and MUM1 expression, supported a diagnosis of DLBCL, non-germinal center subtype.

Treatment

The patient underwent emergent exploratory laparotomy with segmental small bowel resection because of extensive intra-abdominal contamination, bowel perforation, and pelvic abscess formation. Because of significant contamination and hemodynamic instability, temporary abdominal closure was initially performed, with planned staged reconstruction during a subsequent operation. Given the patient’s advanced age, multiple comorbidities, poor functional status, and significant postoperative complications, systemic chemotherapy was deferred.

Outcome and follow-up

The postoperative course was complicated by sepsis, respiratory failure requiring intensive care support, classified as Clavien-Dindo grade IVa, and prolonged ileus, resulting in extended hospitalization. Follow-up imaging demonstrated persistent pelvic lymphoma involvement despite surgical management (Figure 7). Ongoing management discussions focused on supportive care and clinical reassessment in the setting of limited functional reserve and poor tolerance for aggressive systemic therapy.

## Discussion

Primary gastrointestinal lymphomas account for a small proportion of gastrointestinal malignancies, with DLBCL representing the most common histologic subtype. The stomach is the most frequently involved site, whereas primary small intestinal involvement is considerably less common. Among small bowel sites, the terminal ileum is particularly susceptible because of its abundant lymphoid tissue and Peyer’s patches [1-3]. Clinical manifestations are often nonspecific and may include abdominal pain, weight loss, nausea, vomiting, gastrointestinal bleeding, or obstructive symptoms, frequently resulting in delayed diagnosis [4].

Acute bowel perforation is an uncommon but serious presentation of intestinal lymphoma and is associated with significant morbidity and mortality. Proposed mechanisms of perforation include bowel wall infiltration with associated necrosis, ischemic injury, and superimposed infection [5-7]. In many cases, patients present emergently with an acute abdomen, and the diagnosis is established only after surgical resection and histopathologic evaluation. In the present case, the patient presented with pneumoperitoneum and peritonitis requiring emergent laparotomy, clinically mimicking more common causes of bowel perforation, such as ischemic enteritis, diverticulitis, inflammatory bowel disease, or adenocarcinoma.

Histopathologic examination remains essential for the diagnosis and classification of gastrointestinal lymphomas. Diffuse sheets of large atypical lymphoid cells with vesicular chromatin, prominent nucleoli, and brisk mitotic activity are characteristic morphologic findings in DLBCL. Immunohistochemistry plays a critical role in distinguishing lymphoma from poorly differentiated carcinoma and other malignant neoplasms. CD20 positivity confirms B-cell lineage, while additional markers such as CD10, BCL6, and MUM1 assist in subclassification using the Hans algorithm into germinal center B-cell and non-germinal center B-cell phenotypes [8].

Immunophenotypic classification of DLBCL has important prognostic implications. Using the Hans algorithm, the present case demonstrated a non-germinal center B-cell phenotype based on CD10 negativity, BCL6 positivity, and MUM1 positivity. Non-germinal center phenotypes are generally associated with more aggressive clinical behavior and less favorable outcomes compared with germinal center subtypes. Additionally, fluorescence in situ hybridization studies were negative for rearrangements involving MYC, BCL2, and BCL6, with no evidence of high-grade B-cell lymphoma with MYC and BCL2 and/or BCL6 rearrangements (“double-hit lymphoma”) in this case.

Management of primary intestinal DLBCL typically involves systemic chemoimmunotherapy, most commonly rituximab-based regimens such as Rituximab, Cyclophosphamide, Doxorubicin, Vincristine, and Prednisone (R-CHOP). Surgical intervention is generally reserved for complications including perforation, obstruction, hemorrhage, or diagnostic uncertainty [9,10]. In emergent presentations such as bowel perforation with peritonitis, prompt surgical management is required regardless of the underlying etiology; however, recognition of lymphoma remains clinically important because it significantly influences postoperative oncologic evaluation, prognostic assessment, and systemic therapy considerations. Elderly patients with significant comorbidities may have limited tolerance for aggressive systemic therapy, complicating management decisions and adversely affecting prognosis. Complete formal staging was limited in the present case because of the patient’s acute surgical presentation, postoperative clinical deterioration, and poor functional status. Population-based studies have demonstrated that outcomes in DLBCL are influenced by age, disease site, stage, and overall performance status [8].

This case highlights the importance of considering primary gastrointestinal lymphoma in the differential diagnosis of acute abdomen and bowel perforation, particularly in elderly patients. Early recognition and accurate histopathologic classification are essential for appropriate management and prognostic assessment.

Learning points

This case highlights the importance of considering primary gastrointestinal lymphoma in the differential diagnosis of small bowel perforation and acute abdomen, particularly in elderly patients presenting emergently. DLBCL may initially manifest as a life-threatening surgical emergency with bowel perforation before a diagnosis of lymphoma is clinically suspected. Histopathologic examination and immunohistochemical studies remain essential for establishing the diagnosis and accurately subclassifying intestinal lymphomas. Additionally, advanced age, significant comorbidities, and postoperative complications may substantially limit therapeutic options and adversely affect overall prognosis.

## Conclusions

Primary intestinal DLBCL is an uncommon entity that may present as an acute surgical emergency with bowel perforation and peritonitis, particularly in elderly patients. Because the clinical and radiographic findings may mimic more common gastrointestinal conditions, diagnosis often requires histopathologic and immunohistochemical evaluation following surgical resection. Early recognition of this rare presentation is important for accurate diagnosis, prognostic assessment, and timely multidisciplinary management. This case highlights the importance of considering gastrointestinal lymphoma in the differential diagnosis of an acute abdomen and small bowel perforation.
